# Cholangiocarcinoma: Biology, Clinical Management, and Pharmacological Perspectives

**DOI:** 10.1155/2014/828074

**Published:** 2014-02-16

**Authors:** Rocio I. R. Macias

**Affiliations:** ^1^Laboratory of Experimental Hepatology and Drug Targeting (HEVEFARM), University of Salamanca, IBSAL, 37007 Salamanca, Spain; ^2^National Institute of Health Carlos III, CIBERehd, 28029 Madrid, Spain; ^3^Department of Physiology and Pharmacology, Campus Miguel de Unamuno E.D. B-17, University of Salamanca, 37007 Salamanca, Spain

## Abstract

Cholangiocarcinoma (CCA), or tumor of the biliary tree, is a rare and heterogeneous group of malignancies associated with a very poor prognosis. Depending on their localization along the biliary tree, CCAs are classified as intrahepatic, perihilar, and distal, and these subtypes are now considered different entities that differ in tumor biology, the staging system, management, and prognosis. When diagnosed, an evaluation by a multidisciplinary team is essential; the team must decide on the best therapeutic option. Surgical resection of tumors with negative margins is the best option for all subtypes of CCA, although this is only achieved in less than 50% of cases. Five-year survival rates have increased in the recent past owing to improvements in imaging techniques, which permits resectability to be predicted more accurately, and in surgery. Chemotherapy and radiotherapy are relatively ineffective in treating nonoperable tumors and the resistance of CCA to these therapies is a major problem. Although the combination of gemcitabine plus platinum derivatives is the pharmacological treatment most widely used, to date there is no standard chemotherapy, and new combinations with targeted drugs are currently being tested in ongoing clinical trials. This review summarizes the biology, clinical management, and pharmacological perspectives of these complex tumors.

## 1. Primary and Metastatic Liver Cancer

Primary liver cancer accounts for approximately 10–12% of deaths due to cancer. Although the incidence of this group of cancers is lower than 6% of new cancers diagnosed each year worldwide, the prognosis is usually very poor. The most frequent of these tumors are adenocarcinomas, which include hepatocellular carcinoma (HCC) derived from parenchymal cells—accounting for almost 85% of liver adenocarcinomas and cholangiocarcinoma (CCA), derived from biliary epithelial cells and accounting for the remaining 15%. Other rare primary liver tumors include hemangiosarcoma, derived from endothelial cells, and hepatoblastoma, derived from embryonic or fetal hepatocyte precursors. Even less frequent primary liver cancers are fibrosarcoma and lymphosarcoma. It should also be considered that the liver is highly vulnerable to tumor invasion from extrahepatic metastasis. The large size of the liver, its abundant blood supply, and its double-source vascularization explain why it is the second most common seat of metastasis after lymph nodes. Among the tumors that most frequently metastasize to the liver are colorectal cancer, breast cancer, melanoma, and lung cancer.

## 2. Characteristics and Types of CCA 

CCA is not a simple type of tumor; the term refers to a heterogeneous group of malignancies affecting the biliary epithelium. Although, as mentioned above, CCA is much less frequent than HCC, its incidence has increased in western countries over the last few years [1] and it now accounts for about 2% of cancer-related deaths/year worldwide [2]. CCA is characterized by a poor prognosis because its response to chemotherapy is very low and, in most cases, when CCA is diagnosed the tumor is already in a very advanced stage. The reasons for the late diagnosis are the silent evolution of the disease and the fact that its clinical manifestations are nonspecific and mainly related to the biliary obstruction caused by the tumor, such as abdominal pain, pruritus, jaundice, dark urine, clay-coloured stools, or weight loss [3].

Using the criterion of anatomical location, CCAs can be classified (Table 1) as intrahepatic (iCCA) and extrahepatic (eCCA), and these latter differ, depending on their location in the extrahepatic biliary tree, and can be differentiated into distal (dCCA) and perihilar (pCCA). The latter CCAs, frequently located near the confluence of the left and right hepatic ducts, are also known as Klatskin tumours. Although all CCAs share some characteristics, the different sites of formation of the initial tumor affect the patterns of progression and symptoms, as well as the histological features and clinical outcomes. In general, CCA is more often detected in its early stages, when the obstruction and subsequent cholestasis occur due to extrahepatic tumors, but iCCAs may attain large sizes, remaining asymptomatic for a long period before signs of cholestasis appear.

The classic macroscopic classification of intrahepatic tumors (Table 1) includes the mass-forming type, which is the most frequent one and spreads via venous and lymphatic vessels, the periductal-infiltrating type, the intraductal growth type, and the mixed type (mass-forming plus periductal-infiltrating), which is the one with the worst prognosis. Regarding eCCAs (Table 1), these can be of the mass-forming type (nodular), the periductal-infiltrating (sclerosing) type, or the intraductal growth (papillary) type [4]. iCCAs are also classified as well-, moderately, or poorlydifferentiated adenocarcinomas with different degrees of desmoplasia [5].

In an attempt to consider the degree of differentiation, clinical and pathological aspects, genotypes, and even the origin lineage of CCAs in the classification, new categorizations have been proposed [6]. Until recently, CCAs were believed to derive from cholangiocytes, liver stem cells, and peribiliary glands. However, two independent studies performed in rodents [7, 8] have suggested normal hepatocytes as a potential source of CCA, which by neoplastic conversion may transdifferentiate to malignant cholangiolar cells.

Two different categories of iCCA have been described using an integrative genomic analysis: the inflammation class and the proliferation class [9]. Each class has specific activated oncogenic pathways, associated with different clinical outcomes. Shorter survival and earlier recurrence have been observed in patients with proliferation-class iCCAs [9].

The extent of the tumor at the time of diagnosis is a key point for choosing the best treatment for a patient and for assessing the prediction of the prognosis. The TNM Classification of Malignant Tumors of the American Joint Committee on Cancer (AJCC) and the International Union of Cancer Control (IUCC) is the staging system most widely used among oncologists. This system takes into account the degree of invasion of the primary tumor (T1–T4) and the absence or presence of metastasis in regional lymph nodes (N0 or N1) or in distal organs (M0 or M1). The 7th edition of the AJCC Cancer Staging Manual [10] contains for the first time a TNM-staging system for iCCAs (Table 2), which were previously classified as HCCs, and separates extrahepatic bile duct tumors into perihilar (Table 3) and distal (Table 4) tumors, further changing the definitions of the TNM classifications.

For iCCA, the staging considers the presence of single or multiple tumors, vascular invasion, the number of lymph nodes affected by metastasis, and the detection of distal metastasis, but not the tumor size [11], as predictors of adverse outcome.

Since until recently pCCAs and dCCAs were classified in the same group, there is only one recent retrospective, single-institution study carried out in Germany that reports that the new classification for pCCAs represents the severity of the disease and the prognostic value more accurately than the previous staging system [12]. In an Italian study, the same conclusion has been reached for the new iCCA staging system, suggesting that the new classification permits patients to be stratified in the distinct prognostic groups more accurately [13].

## 3. Epidemiology of CCAS

Epidemiological studies have revealed a significant variability in prevalence among different geographic areas and ethnic groups, Asia being the region with the highest prevalence and Australia the geographical area with the lowest prevalence [2]. In the United States, the highest prevalence adjusted by age is found in the Hispanic population (*≈*1 : 100,000), whereas the lowest is found in African Americans (0.17–0.50/100.000) [14]. Mortality is slightly higher in men (1.9/100.000) than in women (1.5/100.000) [15], and the average age of the patients at the time of CCA diagnosis is 70–80 years, except in patients with bile duct cystic disorders, which usually develop CCA much earlier, between 30 and 40 years [16].

A study carried out in the USA on 564 patients reported that eCCA accounted for 90% of CCA cases (pCCA *≈* 50% and dCCA *≈* 40%), whereas iCCA accounted for the remaining 10% [17]. The median survival times after the resection of intrahepatic, perihilar, and distal tumors were 30, 25, and 80 months, respectively [17].

In Europe, the number of deaths due to iCCA has increased over the past few years [18], mainly in western countries [1, 19]. In contrast, the mortality due to eCCA has remained relatively constant and in fact a trend towards decreased rates has been found in most countries [20, 21]. This has been associated with several factors, such as earlier detection, due to the development of more powerful imaging techniques, improvements in the methods of patient selection, and advances in surgery [22]. In this respect, it should also be considered that some years ago the difficulty involved in carrying out an accurate diagnosis during the early stages could have underestimated the true incidence of iCCA [23]. This deviation in the actual epidemiological values could be corrected in the future if more sensitive and accurate biochemical, genetic, and immunohistological markers were used in the early diagnosis of CCA.

## 4. CCA Risk Factors

Although in approximately 50% of the cases of CCA reported in the literature the presence of the predisposing conditions involved in the development of CCAs could not be clearly identified, there are several well-known risk factors associated with the appearance of these tumors; these are commented on below.

Parasitic infection by *Opisthorchis viverrini* and *Clonorchis sinensis* is responsible for the high incidence of CCA in Asia [24, 25], whereas in developed countries patients with chronic hepatitis C, primary sclerosing cholangitis, cirrhosis, hepatolithiasis, or metabolic syndrome are those with an enhanced risk of developing CCA [26–28].

Strong associations of bile duct cystic disorders (intrahepatic or extrahepatic cysts and Caroli's disease) and CCA have been found, even after the surgical removal of cholecochal cysts [16]. All these conditions share the presence of a certain degree of liver damage due to the chronic inflammation of the bile ducts associated with cholestasis. In fact, chronic cholestasis has often been associated with CCA and it has recently been shown that the accumulation of bile acids in the liver tissue stimulates the development of CCAs. This is not due to a direct carcinogenic effect but to the ability of these molecules to behave as cocarcinogenic agents. Such activity is based on bile acid-induced inflammation, ductular proliferation, and impaired FXR-dependent chemoprotection [29].

Xenobiotics, such as ethanol [26], chemicals such as dioxin or vinyl chloride, or the radiocontrast agent Thorotrast (thorium dioxide), which was extensively used in the 1930s–1940s [30], have also been recognized as risk factors associated with CCA development.

Type 2 diabetes mellitus, obesity, and smoking, as well as an important number of genetic polymorphisms, have been proposed as risk factors for CCA, but these data need to be verified in the future [25].

A close followup of patients at risk of developing this type of tumor would be the recommended best practice to achieve early detection of CCA. However, except for patients with primary sclerosing cholangitis who are already being monitored in some western countries, the rarity of the disease and the large number of predisposing conditions complicate the selection of the target population for inclusion in routine surveillance programs.

## 5. CCA Molecular Pathogenesis

Despite the important efforts made in the field recently, the molecular mechanisms underlying the development of CCA are largely unknown. It has been suggested that chronic cholestasis and inflammation may enhance cell proliferation, which would increase the risk of the accumulation of somatic mutations [31, 32]. In cholangiolar cells, proinflammatory cytokines, such as TNF-*α* and IL-6, stimulate the expression of inducible nitric oxide synthase (iNOS), enhancing NO production. Reactive oxygen species, together with NO, interact with DNA and inhibit DNA repair mechanisms. The result is the promotion of mutagenesis [33]. In addition, NO and several cytokines can inhibit cholangiocyte apoptosis, both directly, by the nitrosylation of caspase 9, and indirectly, through the stimulation of cyclooxygenase 2 (COX-2), the rate-limiting enzyme in prostaglandin biosynthesis. Via prostaglandin E2 production, this enzyme is able to inhibit apoptosis and activate the cell cycle [34].

In experimental models of chemically induced CCA in rats a significant increase in the expression of IL-6 has been found in tumor cells [29]. Moreover, IL-6 has also been found to be elevated in the serum of patients with CCA [35]. This cytokine is known to play a key role in cholangiocyte malignization. First, IL-6 favors the ability of these cells to elude apoptosis by upregulation of the antiapoptotic protein Mcl-1 (myeloid cell leukemia-1) through the STAT3 and AKT signaling pathways [36, 37]. Second, IL-6 activates mitogen-activated protein kinase p38 [38], which promotes cell proliferation and stimulates telomerase activity, which reduces senescence in malignized cholangiocytes [39].

COX-2 can be activated by members of the EGFR (epidermal growth factor receptor) family, in particular the tyrosine kinase ERBB2 (HER-2/neu) [40]. This is overexpressed in a moderate proportion of CCAs, mostly of the eCCA type [41, 42], as well as in animal models of cholangiocarcinogenesis [29, 43]. Moreover, a high ERBB2 expression has also been associated with increased invasiveness, proliferation, and mobility of CCA cells [44].

Previous ‘‘*in vitro*” studies have suggested an indirect mutagenic ability of most hydrophobic bile acids, such as deoxycholic acid, which may favor cholangiocarcinogenesis. It has been reported that this effect could be due to EGFR pathway-dependent upregulation of COX-2 [45]. However, recent studies have shown that bile acids do not induce direct damage in DNA [29] but act as promoters, stimulating cholangiolar cells proliferation, probably via the activation of growth factors, such as EGFR.

Furthermore, it should be noted that the membrane receptor TGR5, which responds to bile acids, is overexpressed in CCAs and confers resistance to apoptosis [46]. In contrast, the nuclear receptor FXR, which also behaves as a bile acid sensor, seems to play a role in the protection against the development of CCA [47]. Thus, FXR-knockout mice spontaneously develop liver tumors (HCC and CCA) [48, 49].

The expression of the vascular endothelial growth factor-C (VEGF-C), an important lymphangiogenetic factor, has been found elevated in approximately 50% of CCAs analysed [50]. Interestingly, VEGF-C upregulation was associated with a worse prognosis in patients with iCCA [50]. The activation of VEGF receptor (VEGFR) stimulates the proliferation and migration of endothelial cells, and these effects are enhanced by estrogens, through the induction of the expression of VEGFR [51].

The MET receptor is also overexpressed in CCA [52]. By triggering the activation of several routes of intracellular signaling, the binding of its ligand HGF to MET stimulates the migration and invasion of CCA cells [53].

## 6. CCA Diagnosis

The clinical history, together with radiological and pathological analyses, of patients is used to distinguish CCA from other entities that may be misdiagnosed as such. These include HCC, metastatic pancreatic cancer, and gallbladder cancer [5]. The diagnosis of CCA is often difficult, which complicates patient management.

Magnetic resonance imaging (MRI) is the radiologic mode of choice [54] for visualizing the location and the extent of biliary disease. The use of gadolinium, which accumulates in neoplastic liver tissue, improves the detection capacity of MRI [55]. The different pattern of the uptake and washout of the contrast agent helps to distinguish between HCC and CCA, at least when tumors are larger than 2 cm [56]. Computed tomography (CT) permits the visualization of liver parenchyma, biliary dilatation, lymph nodes, and intrahepatic tumors and extrahepatic metastases. Moreover, CT may be more accurate in the prediction of resectability [57]. Ultrasonography permits the identification of bile duct dilatation proximal to the obstructive mass and is mainly used to detect pCCA. In contrast, iCCA is difficult to detect with this technique [58]. Fluorodeoxyglucose-positron emission tomography (FDG-PET) is often useful for identifying distant metastases that cannot be detected by other techniques but adds little when the other techniques have been used successfully [59]. Endoscopic retrograde cholangiography (ERC) is useful in the diagnosis of pCCA to identify strictures in bile ducts, although it is not possible to distinguish between malignant and benign lesions [60].

Advances in the understanding of the development of CCAs have prompted researches to find new markers that will permit early detection, which—owing to their silent evolution and late diagnosis [61]—is particularly important in the case of these tumours.

The serum tumor markers CA19-9 (carbohydrate antigen 19-9) and CEA (carcinoembryonic antigen) are useful in diagnosis and monitoring during and after the treatment of gastrointestinal malignancies and are included in routine clinical tests due to their relatively low cost. For CCA, the predictive value of CA19-9 seems to be higher than that of CEA and should be used together with other diagnostic techniques [62]. CA19-9 serum values >129 U/mL in patients with primary sclerosing cholangitis reflect a sensitivity of 79% and a specificity of 98%; however, the usefulness of this tumor marker when CCA is not associated with primary sclerosing cholangitis is very low [63].

Despite the efforts to identify CCA-specific markers in serum and bile, none of them proposed to date (mucins 1 and 5AC, metalloproteinases 7 and 9, claudin-4, IL6, IGF1, cytokeratin 19 fragments, etc.) has reached a level of specificity and sensitivity adequate for recommendation as useful tools in clinical practice [64, 65].

There is little consensus regarding the usefulness of carrying out liver biopsies for the diagnosis of CCA because on one hand there is a serious risk of spreading the tumor and causing haemorrhages and on the other hand, at least in the case of iCCAs, the results of the histopathology analyses are usually not definitive. Nevertheless, it is generally accepted that the biopsy is necessary in patients with cirrhosis because, in these cases, the radiological techniques do not permit a distinction between small HCC and CCA.

Histochemical and immunohistochemical analyses with specific antibodies against cytokeratin-7 (CK-7) and CK-19 permit the confirmation of diagnosis after resection and provide useful prognostic information.

## 7. Treatment of CCA

The options for the treatment of CCA are limited and associated with high rates of perioperative mortality, recurrence, and short survival times. Surgical resection of tumors with negative margins is the best option for all subtypes of CCA, although it is only achieved in less than 50% of cases, and it is often necessary to perform a partial hepatectomy together with the removal of regional lymph nodes. Curative resection, or resection of tumor-free surgical margins (R0), remains the best chance for long-term survival, and lymph node status is the most important prognostic factor following R0 resection [17]. Routine lymphadenectomy at the time of surgical resection has been proposed in order to increase the chance of survival; however it can be omitted in patients with solitary, small peripheral CCA because the probability of lymph node metastasis is very low [66].

In iCCAs, resection has usually been indicated in patients with a solitary tumor and with no underlying hepatic disease. The best prognostic factors are R0 resection without lymph node invasion, while tumor diameter, histology, and differentiation are poor predictors of good outcome. The 5-year survival rates reported in the past few years vary from 20 to 60% [17]. A recent study has concluded that major hepatectomy for iCCA is also indicated in selected cirrhotic patients because the overall morbidity, hospital mortality rates, and the appearance of liver failure and other complications (superficial wound infection, abscesses, sepsis, pancreatic leakage, delayed gastric emptying, or biliary leakage) are similar in patients with and without cirrhosis [67].

Resection is a suitable treatment option for extrahepatic tumors, depending on the extent in the biliary tree and hepatic vasculature. When such tumors are restricted to one lobe, there is no metastasis, and liver function is preserved, resection is recommended. Partial hepatectomy is the only factor associated with better outcome, probably because this option permits negative margins to be achieved. The 5-year survival for R0-resected eCCAs is about 30% [17], with recurrence observed in the majority of patients due to disseminated tumors or the *de novo* formation of tumors in the already oncogenic liver tissue. Thus, surgical resection is not recommended for CCAs in patients with primary sclerosing cholangitis because the recurrence rate is very high, close to 90%.

Liver transplantation is usually recommended for patients with pCCA diagnosed in the early stages, which cannot be removed surgically, and when no metastases are detected [68] and also for patients with tumors developed in livers with reduced function or underlying a biliary inflammation pathology, such as primary sclerosing cholangitis. Liver transplantation performed after neoadjuvant chemoradiation in selected patients, due to organ shortage, has afforded a very good disease-free 5-year survival (>80%), providing a better outcome and fewer recurrences than conventional resection [69, 70].

Tumor ablation performed percutaneously with sonographic guidance using radiofrequency or microwave energy can offer efficient therapy for nonoperable tumors up to 5 cm in size. Complete tumor destruction without local recurrence was reached in 85% of patients with iCCA, with a median overall survival period of 38.5 months, while major complications occurred in 6% of the cases [71].

The currently available adjuvant therapies, chemotherapy, and radiotherapy have not been shown to improve the outcome or time to recurrence of patients when administered either before or after surgery, although no large randomized trials have been conducted. It has even been reported that radiation therapy may elicit unwanted results, such as difficulties in handling cholangiopathies [4].

Palliative chemotherapy, radiotherapy, and photodynamic therapy have been relatively ineffective in treating non-operable CCAs, with a 5-year survival <5% without resection, due to the refractoriness of these tumors.

For some patients with non-operable tumors, biliary drainage through a tiny metal or plastic tube (‘‘biliary stent”) may result in an improvement of the patient's situation due to relief of the obstructive cholestasis. This can be done percutaneously, although with these external drainage systems patients may experience certain discomfort, and it is the only option in cases of complete biliary obstruction. Stents may eventually cease to function because of tumor overgrowth, obstruction, or other reasons; plastic stents need to be changed every 3 months, while metal stents can be maintained for longer times [72]. Cholestasis is a risk factor for hepatic failure after liver resection and stents are now widely used for preoperative drainage. Self-expanding metal stents are preferred because they provide rapid biliary decompression and a reduced complication rate after insertion [73].

Evaluation of the clinical usefulness of other therapeutical strategies that have emerged in recent years requires further investigation. Thus, photodynamic therapy seems to relieve pain, improves the flow of bile through the biliary tree, and increases survival.

Transarterial chemoembolization (TACE), which increases the local concentration of chemotherapeutic agents and reduces systemic exposure [74], has shown promising results, increasing survival [75], and radioembolization [76] also seems to increase survival. Thus, these regional therapies are considered as an option for treating small tumors when the general health condition of the patient does not permit a more aggressive treatment.

An important number of phase-II clinical trials have been carried out with different chemotherapy regimes to treat CCA, using single or combined agents (Table 5). In contrast, to date the number of phase-III trials has been low. These studies have some limitations, mainly due to the heterogeneity of the tumor types included (grouped as biliary tract cancer in some studies, or CCA without separation between types), different extents of the disease, naïve patients mixed together with patients who have previously received different therapies, small numbers of patients included, and so forth. This has contributed to the fact that, even though the moderate benefits and tolerability of some regimes have been described, as commented below, no standard treatment for CCA has yet been established.

5-Fluorouracil (5-FU)-based regimens were among the first reported in biliary tract cancers, together with uracil-tegafur and S-1, which is a combination of tegafur/gimeracil/oteracil potassium. Compared with the median survival of untreated patients with advanced CCA, which was only 3.9 months, all of them obtained a moderate response and only S1 was well tolerated by the patients [81, 98].

The usefulness of gemcitabine alone, or gemcitabine-based combination regimes (with cisplatin derivatives, capecitabine, or S-1), has been reported by the authors of several clinical trials, with variations regarding the response and overall survival rates (Table 5). In fact, for some time gemcitabine has been the first-line chemotherapeutic agent recommended for biliary tract carcinomas in Japan [99]. The gemcitabine plus capecitabine regime is well tolerated by CCA patients and survival is slightly better than with gemcitabine alone (Table 5) [82–85]. Some improvements have also been obtained with regimens based on gemcitabine combined with cisplatin or its derivatives [87–91].

Two randomized clinical trials conducted in the UK and Japan demonstrated that the combination of gemcitabine plus cisplatin provided better survival benefits than treatment with gemcitabine alone [93, 94]. Nonetheless, success has been very poor because this combination therapy increased survival by only 3–5 months with respect to the administration of gemcitabine alone.

Importantly, the success of these regimes also depends on the performance status (PS) of patients with nonresectable biliary tract adenocarcinomas (gallbladder, iCCA, eCCA, and ampullar) [89]. In fact, in patients treated with gemcitabine plus oxaliplatin (GEMOX) with a PS of 0–2, the median response rate was 35.5%, with a median overall survival of 15.4 months, while in patients with a PS >2 the median response rate was reduced to 22% and overall survival to 7.6 months.

The triplet of drugs included in the GEMOX and capecitabine regime has recently been assayed in patients with advanced CCA [97]. The median response rate was 34% and median survival 12.5 months. Although no data concerning the response of each type of tumor are available, the promising results suggest that this combination should be explored in a randomised phase-III multinational study.

Currently, gemcitabine plus cisplatin, or more frequently GEMOX, is the basis for new combined therapies included in new clinical trials whose results are expected to permit the establishment of a standard treatment for CCA in the near future.

## 8. CCA Pharmacological Perspectives

**Figure 1:**
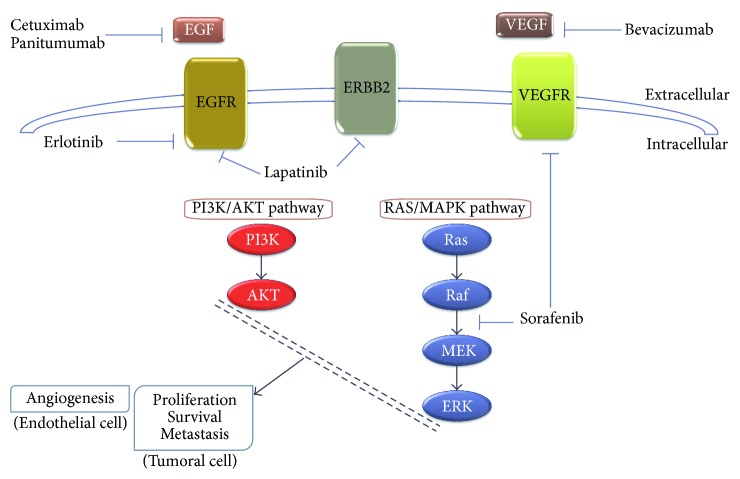
Main signalling pathways (PI3K/AKT and RAS/MAPK) activated in cholangiocarcinogenesis by activation of tyrosine kinase receptors, such as EGFR, ERBB2, VEGFR, and others, and molecular mechanism of action of targeted therapies. In tumoral cells, the activation of signalling pathways induces the transcription of genes involved in proliferation, survival, and cell growth, while in endothelial cells the activation of these pathways stimulates angiogenesis.

Since the available chemotherapy provides minimal benefit in the treatment of CCA, considerable efforts are being invested in developing new therapeutic strategies to treat these patients. Among them the use of targeted therapies based on the expression of growth factors (Figure 1) and activation of signal transduction pathways that play important roles in tumor cell proliferation, progression, and invasiveness should be mentioned.

An interesting family of drugs is formed by the tyrosine kinase receptor inhibitors (TKIs), involved in signaling pathways for cell survival and angiogenesis. Some of these drugs are still under investigation, while others have already been incorporated into the pharmacological treatment of various cancers, including CCA (Table 6). Among these drugs is sorafenib, a multikinase inhibitor that blocks VEGFR and platelet-derived growth factor receptor (PDGFR), and the RAF serine/threonine kinases along the RAF/MEK/ERK pathway. By inhibiting these kinases, genetic transcription involving cell proliferation and angiogenesis is inhibited. Sorafenib has a modest effect on HCC [100] and other tumors, while its effect on CCA has been reported to be very weak [101] or weak [102]. A marginal response has also been observed with the VEGFR inhibitor sunitinib as second-line treatment for patients with unresectable metastatic CCA [103], and a moderate increase in overall survival and no toxicity have been reported for selumetinib, an inhibitor of mitogen-activated protein kinases 1 and 2 (MEK1/2) [104].

One of the characteristics of CCA is the over-expression of EGFR [113], which has been associated with enhanced cell proliferation. Indeed, the overexpression of this gene may cause a more aggressive phenotype, but it can also render the tumor more sensitive to EGFR antagonists, such as erlotinib and the monoclonal antibody against EGF cetuximab, which have proved to be effective in reducing proliferation *in vitro* using CCA-derived lines [114]. In *in vivo* assays, a more effective strategy has been the use of two inhibitors, such as NVP-AEE788, which inhibits EGFR and ERBB2 [115], and vandetanib, an inhibitor of EGFR and VEGFR [116].

The results of a phase-II study of erlotinib in patients with advanced biliary tract cancer (iCCA, eCCA, and gallbladder cancer) suggested a potential benefit in survival [105], which prompted the use of this drug in combination with other targeted agents to enhance efficacy, such as bevacizumab, a VEGF inhibitor [107], which did not improve the benefits of erlotinib administered alone. A phase-III clinical trial has evaluated the addition of erlotinib to conventional chemotherapy GEMOX, but no improvement in survival was observed on comparing GEMOX alone and the combination with erlotinib [111].

A phase-II study of lapatinib, an inhibitor of EGFR and ERBB2, in patients with liver cancers revealed no response in CCA and a very low one in HCC (5%) [106]. Two clinical trials with GEMOX plus the EGF inhibitor cetuximab [108] or plus bevacizumab [109] reported an increase in the response rate and the overall survival, with good tolerance. Similar results were obtained with GEMOX/capecitabine + another EGF inhibitor, panitumumab [110]. Finally, a recent clinical trial with 39 patients with advanced CCA treated with gemcitabine/cisplatin plus sorafenib reported similar efficacy but higher toxicity as compared with previous studies without sorafenib [112].

One of the transport systems involved in the uptake of cationic inhibitors of tyrosine kinase receptors by healthy and tumor liver cells is OCT1 (organic cation transporter 1). Two recent publications have reported that a marked decrease in the expression of this transporter in tumor tissue occurs in both HCC and CCA [117, 118]. This may limit the activity of sorafenib in these tumors. Furthermore, the presence of aberrant genetic variants partially or completely abolished the ability of tumor cells to take up the drug through this route, which may markedly determine the response of the tumor to treatment with sorafenib [119]. These findings suggest that an appropriate selection of CCA patients suitable for treatment with sorafenib is crucial.

Transporters that account for the uptake and efflux of endogenous compounds across the basolateral and apical membranes of hepatocytes and cholangiocytes are also involved in the sensitivity and refractoriness to pharmacological treatment of liver tumours (for a review see [120]). In fact, another strategy to target drugs to cholangiolar cells is to take advantage of the presence of specific plasma membrane transporters in these cells, such as the bile acid transporter ASBT [121]. Our group has synthesized and characterized several members of a new family of compounds designated ‘'BAMETs” (from bile acid and METal) by binding bile acids to cisplatin or other metals [122–124]. These compounds with liver-targeting properties exert a strong cytostatic activity against liver tumors [125, 126] while maintaining bile acid organotropism [127], thus reducing side effects in extrahepatic tissues [126].

Recent preliminary results suggest that the cytostatic bile acid derivative Bamet-UD2, synthesized by the conjugation of ursodeoxycholic acid to cisplatin, is efficiently taken up by cholangiolar tumor cells, and it has been shown to inhibit tumor growth in both *in vitro* and *in vivo* models [128].

Owing to the importance in the CCA of the development of signalling pathways involving MET and COX-2, there is a reasonable hope regarding the results that will eventually be obtained using MET and COX-2 inhibitors. This strategy is currently being tested in several preclinical and clinical trials in other tumors, such as tivantinib in HCC [129], cabozantinib in advanced thyroid cancer [130], and COX-2 inhibitors such as celecoxib, which inhibits the proliferation of CCA cells *in vitro* [131] and reduces tumor growth in rats with chemically induced CCA [132].

There are several ongoing clinical trials addressing CCA (see information from http://clinicaltrials.gov/) to investigate drugs as monotherapy: everolimus, or several combined therapies: GEMOX/panitumumab, gemcitabine/cisplatin/selumetinib, gemcitabine/irinotecan/panitumumab, gemcitabine/capecitabine/bevacizumab, 5-FU/leucovorin/oxaliplatin/cediranib, GEMOX/erlotinib, sorafenib/erlotinib, and S-1/abraxane.

## 9. Conclusions 

The increasing worldwide incidence of CCAs (with half the patients developing the disease with no association with known risk factors), the difficulty involved in early diagnosis because the symptoms (nonspecific) do not appear until tumor development has reached an advanced stage, our incomplete knowledge of the pathogenesis of CCAs, the low efficacy of available pharmacological treatments, and the lack of biomarkers that permit the diagnosis and/or identification of responders to treatments are the main challenges that scientists must face in forthcoming years.

The new classification of CCAs, depending on the location of tumors in the biliary tree, and all the efforts aimed at understanding the molecular basis of CCA, together with the identification of new targeted therapies and ongoing clinical trials, will hopefully allow well-established and more effective therapeutic options to be offered to patients suffering from this complex type of liver tumor.

## Figures and Tables

**Table 1:** Classification of cholangiocarcinomas (CCA).

Anatomical location		Macroscopic growth pattern
Intrahepatic (iCCA)		Mass-forming Periductal-infiltrating Intraductal Mixed (mass-forming + periductal-infiltrating)

Extrahepatic (eCCA)	(i) Perihilar (Klatskin) (ii) Distal	Mass-forming (nodular) Periductal-infiltrating (sclerosing) Intraductal (papillary)

**Table 2:** TNM staging system for iCCAs (7th edition).

Stage	Tumor	Node	Metastasis
I	T1	N0	M0
II	T2	N0	M0
III	T3	N0	M0
IVA	T4	N0-N1	M0
IVB	T1–T4	N0-N1	M1

T1: solitary tumor without vascular invasion; T2: solitary tumor with vascular invasion or multiple tumors with/without vascular invasion; T3: tumor or tumors perforate visceral peritoneum or local hepatic structures; T4: tumor with periductal invasion.

N0: no regional lymph node metastasis; N1: regional lymph node metastasis.

M0: no distant metastasis; M1: distant metastasis.

**Table 3:** TNM staging system for pCCAs (7th edition).

Stage	Tumor	Node	Metastasis
0	Tis	N0	M0
I	T1	N0	M0
II	T2a, 2b	N0	M0
IIIA	T3	N0	M0
IIIB	T1–T3	N1	M0
IVA	T4	N0-N1	M0
IVB	T1–T4	N0-N1	M1

Tis: carcinoma *in situ*; T1: tumor confined to the bile duct, with extension up to muscle layer or fibrous tissue; T2a: tumor invades surrounding adipose tissue; T2b: tumor invades adjacent hepatic parenchyma; T3: tumor invades unilateral branches of portal vein or hepatic artery; T4: tumor invades main portal vein or hepatic artery or bilateral branches.

N0: no regional lymph node metastasis; N1: regional lymph node metastasis.

M0: no distant metastasis; M1: distant metastasis.

**Table 4:** TNM staging system for dCCAs (7th edition).

Stage	Tumor	Node	Metastasis
IA	T1	N0	M0
IB	T2	N0	M0
IIA	T3	N0	M0
IIB	T1–T3	N1	M0
III	T4	N0-N1	M0
IV	T1–T4	N0-N1	M1

T1: tumor confined to the ductal wall; T2: tumor beyond the ductal wall; T3: tumor invades adjacent organs; T4: tumor invades celiac axis or superior mesenteric artery.

N0: no regional lymph node metastasis; N1: regional lymph node metastasis.

M0: no distant metastasis; M1: distant metastasis.

**Table 5:** Phase-II or -III clinical trials with conventional chemotherapy in patients with unresectable CCA.

Treatment	Patients with biliary cancer	Well-diagnosed CCA patients^a^	Response rate (%)	Median OS (months)	References
GEM	32	22	22	11.5	[77]
GEM	30	30	30	14	[78]
GEM	40	12	17.5	7.6	[79]
CAP	26	18	6	8.1	[80]
S-1	40	15	35	9.4	[81]
GEM + CAP	45	23	31	14	[82]
GEM + CAP	44	30	32	14	[83]
GEM + CAP	12	11	17	14	[84]
GEM + CAP	52	35	13	7	[85]
GEM + S-1	35	20	34.3	11.6	[86]
GEM + cisplatin	40	39	27.5	8.4	[87]
GEM + cisplatin	29	19	34.5	11	[88]
GEM + oxaliplatin	33	20	35.5	15.4	[89]
GEM + oxaliplatin	31	21	26	11	[90]
GEM + oxaliplatin	53	32	18.9	8.3	[91]
GEM vs	32	18	5.6	15	[92]
GEM + S-1	30	14	7.1	9.5	[92]
GEM vs	206	119	15.5	8.1	[93]
GEM + cisplatin∗	204	122	26.1	11.7	[93]
GEM vs	42	25	11.9	8	[94]
GEM + cisplatin∗	41	22	19.5	13	[94]
GEM + 5-FU + leucovorin	42	24	12	9.7	[95]
Irinotecan + oxaliplatin	28	28	17.9	9.2	[96]
GEMOX + CAP		41	34	12.5	[97]

5-FU: 5-fluorouracil; CAPE: capecitabine; GEM: gemcitabine; GEMOX: gemcitabine + oxaliplatin; OS: overall survival; S-1: tegafur + gimeracil + oteracil potassium.

^a^Patients with CCA (intrahepatic or extrahepatic) out of the total of patients included as suffering from biliary tract cancer in the clinical trial.

^*^Phase-III clinical trial.

**Table 6:** Clinical trials with targeted therapies in patients with CCA.

Treatment	Patients with biliary cancer	Well-diagnosed CCA patients^a^	Response rate (%)	Median OS (months)	References
Erlotinib		24 of 42	8	7.5	[105]
Lapatinib		9 of 17	0	5.2	[106]
Sorafenib		32 of 46	2.2	4.4	[101]
Sorafenib		19 de 31	0	9	[102]
Selumetinib		17 of 28	12	9.9	[104]
Sunitinib		41 of 56	8.9	4.8	[103]
Erlotinib + bevacizumab		43 of 53	12	9.9	[107]
GEMOX + cetuximab		27 of 30	63	11.6	[108]
GEMOX + bevacizumab		25 of 35	40	12.7	[109]
GEMOX + capecitabine + panitumumab		38 of 46	33	10	[110]
GEMOX vs	133	84	16	9.5	[111]
GEMOX + erlotinib	135	96	30	9.5	[111]
Gemcitabine + cisplatin + sorafenib		39	50	14.4	[112]

GEMOX: gemcitabine + oxaliplatin; OS: overall survival.

^a^Patients with CCA (intrahepatic or extrahepatic) out of the total number of patients included as suffering from biliary tract cancer in the clinical trial.
